# Institutional Surveys and the Patient Feedback Mechanism in a Romanian Public Emergency Hospital: A Longitudinal Comparative Analysis, 2019–2024

**DOI:** 10.3390/healthcare14131835

**Published:** 2026-06-24

**Authors:** Mihaela-Denisa Coman, Dan-Marius Coman, Petronela-Alice Grigorescu

**Affiliations:** 1 Institute of Multidisciplinary Research for Science and Technology, Valahia University of Târgoviște, 130004 Târgoviște, Romania; denisa.coman@valahia.ro; 2 Doctoral School of Economics and Humanities, Valahia University of Târgoviște, 130004 Târgoviște, Romania; alice.grigorescu@valahia.ro

**Keywords:** patient satisfaction, hospital quality, longitudinal study, Romania, patient experience gap

## Abstract

**Highlights:**

**What are the main findings?**

- Institutional satisfaction surveys at SJUT consistently recorded satisfaction scores between 96.88% and 97.45% across 2019–2024, while an independent national instrument, the Patient Feedback Mechanism (MFP), recorded satisfaction with medical services between 70.7% and 88.9% over the same period for the corresponding item. The gap between the two sources ranged from approximately 8 to 27 percentage points and was statistically significant in every year (*p* < 0.001).
- The gap did not follow a steady trend. MFP satisfaction with medical services declined from 89.9% in 2019 to a low of 70.7% in 2021, recovered to 75.8% in 2023, and settled at 74.8% in 2024 (Mann–Kendall *p* = 0.469, non-significant). A separate MFP item asking whether respondents would report a request for money from medical staff remained in a narrow 4.0–5.5% band between 2019 and 2023 (Mann–Kendall *p* = 0.82 for this sub-period), with no significant trend; a higher value (6.9%) was recorded in 2024 using a redesigned version of this item introduced nationally in February 2024, which is not directly comparable to earlier years (Section 2.5).

**What are the implications of the main findings?**

- The standardised institutional questionnaire administered at discharge cannot detect the dimension covered by MFP Item 8 (willingness to report a money request from staff), which has no equivalent in the institutional questionnaire and is present, at a non-trivial rate, in every year studied. This means hospital management can record satisfaction scores above 96% while a separate, nationally available data source documents a dimension of institutional integrity that is otherwise invisible to routine monitoring, even though the available data do not support a clear rising trend over 2019–2023.
- All eight MFP items, including cleanliness and self-reported health status, follow a broadly similar trajectory over the six years: a decline through 2021, followed by partial recovery in 2022–2023. This similarity suggests that the SMCSP-MFP gap and its temporal pattern reflect the conditions of administration and the period studied, rather than being confined specifically to medical-service satisfaction. Hospital managers can use existing MFP data, already collected at the national level, as a low-cost complement to Quality and Patient Safety Management Service (SMCSP) monitoring, with particular attention to items that are collected but not reported as standalone SMCSP indicators.

**Abstract:**

**Background/Objectives**: Standardised institutional patient satisfaction surveys are the primary quality-monitoring tool in Romanian public hospitals, but their ability to capture the full range of patient experiences remains uncertain. This study quantifies the discrepancy between institutional patient satisfaction scores and an independent, unmediated national feedback instrument, the Patient Feedback Mechanism (MFP), at Targoviste County Emergency Hospital (SJUT) over a six-year period (2019–2024), and examines item-level MFP results across eight dimensions of the patient experience, including dimensions not captured by the institutional indicators routinely reported by SMCSP. **Methods:** A sequential design combined six years of institutional satisfaction data (2019–2024) from SJUT (N = 32,176 questionnaires) with item-level MFP results for the same period, covering eight questions on medical services, cleanliness, out-of-pocket medication costs, staff involvement, communication, recommendation intent, self-reported health outcome, and willingness to report requests for money from staff. Hypotheses were tested using two-proportion z-tests with Wilson confidence intervals, Mann–Kendall trend analysis, and Cohen’s h for effect sizes. **Results**: Institutional satisfaction remained consistently high (96.88–97.45%), while MFP satisfaction with medical services ranged from 70.7% to 88.9% across the same years, yielding gaps of 7.9 to 26.7 percentage points, significant in every year (*p* < 0.001; Cohen’s h ranging from 0.32 to 0.82). The gap did not follow a monotonic trend (Mann–Kendall *p* = 0.469); instead, it widened to a peak in 2021 and narrowed progressively through 2024. A parallel comparison between the Quality and Patient Safety Management Service (SMCSP) overall impression item (exceeding 99%) and the MFP recommendation item (69.9–76.3%) showed even larger gaps, of 23.3 to 29.6 percentage points. The MFP item on willingness to report requests for money from staff, which is not part of SMCSP’s reported institutional indicators, remained in a narrow 4.0–5.5% range between 2019 and 2023 with no significant trend (Mann–Kendall *p* = 0.82); a higher 2024 value (6.9%) coincides with a national redesign of this item and is not directly comparable to earlier years. **Conclusions**: Institutional surveys and an independent national feedback instrument offer structurally distinct perspectives on hospital performance, reflecting differences in administration rather than equivalent estimates of patient satisfaction. The discrepancy between sources is significant and persistent, though not monotonic, widening sharply during 2021 before narrowing. One item with no institutional equivalent documents a measurable, non-trivial proportion of patients willing to report informal payment requests every year, although the available data do not establish whether this proportion is rising over time. Systematic use of existing MFP data, already collected nationally, can complement institutional surveys at minimal additional cost, provided the two instruments are interpreted as structurally different rather than as alternative estimates of the same quantity.

## 1. Introduction

Measuring patient satisfaction is a fundamental component of quality governance in public hospitals across Europe. Satisfaction data inform key managerial decisions, resource allocation, and public accountability mechanisms, including comparative reporting between units and performance-based funding [1,2]. In Romania, as in other systems with universal public funding, hospitals are required by regulation to periodically collect satisfaction data through standardised questionnaires administered internally by each unit’s SMCSP. These results are reported to accreditation authorities in accordance with national quality management legislation.

Standardised institutional questionnaires have made considerable methodological progress over the past four decades, evolving from the original SERVQUAL instruments to more recent patient-centred formats [3,4,5]. However, the theoretical framework for these tools, largely based on the structure–process–outcome model [6], faces persistent criticism in the literature. Scholars point to structural limits such as the predefinition of assessed dimensions, the risk of social desirability bias, and ceiling effects in populations with low expectations or limited alternatives [7,8,9,10]. Even early meta-analytic evidence showed that what patients value in their care does not always align with what standardised instruments ask them about, a concern that remains central to the predefinition problem described above.

The limitations of institutional instruments are not merely theoretical. Standardised questionnaires are built around predefined dimensions, which makes them insensitive to concerns that patients themselves consider important, but that fall outside the established categories. The ceiling effect compounds this problem: populations with limited access to alternatives and low expectations tend to report high satisfaction even in objectively unsatisfactory conditions [10,11]. Recent methodological work comparing response formats in public hospital settings confirms that ceiling effects and acquiescence bias remain a persistent measurement challenge for patient satisfaction questionnaires [12].

Social desirability bias adds a further layer of distortion when patients complete questionnaires in the presence or proximity of medical staff, producing responses that are systematically more positive than those obtained through anonymous surveys administered after discharge [9,10,13]. More recent national instruments, such as HCAHPS in the United States or the NHS Friends and Family Test in the United Kingdom, have attempted to address some of these shortcomings by standardising data collection at scale and enabling inter-institutional comparison [5,14], but the structural blind spots of predefined formats remain.

A different kind of response to these limitations comes from instruments designed to be completed independently of the treating unit, after discharge, without staff involvement. Compared with discharge-administered questionnaires, such unmediated instruments are expected to reduce social desirability effects associated with staff-mediated completion [9,10,12], at the cost of substantially lower and self-selected response rates. Evidence from large-scale randomised survey-mode experiments confirms that the channel through which a satisfaction instrument is administered can materially affect both response rates and the resulting scores [15].

In Romania, this approach was formalised in December 2016 through the Patient Feedback Mechanism (MFP), a national digital system through which discharged patients evaluate their inpatient experience via SMS or web form, independently of the treating unit [16]. National-level analyses of MFP data have documented both a substantial correction in reported satisfaction following the shift from paper-based, staff-mediated collection to unmediated digital collection, and persistently low participation rates across most public hospitals [17,18]. These national findings raise a hospital-specific question that, to date, has not been addressed for any individual Romanian public hospital: does the gap between an institutional, discharge-administered questionnaire and the unmediated MFP appear at the level of a single unit, and if so, what does it look like over time and across the specific dimensions covered by the MFP instrument?

In the Romanian context, institutional satisfaction scores are consistently high [19,20], yet these are not always supported by independent evaluations using a different administration mode. A persistent gap between a discharge-administered questionnaire and an unmediated national instrument may indicate dimensions of the patient experience that remain invisible to institutional monitoring, including out-of-pocket costs, perceived communication quality, and informal payment requests. This has direct implications for the UN Sustainable Development Goals, specifically SDG 3 on health quality and SDG 16 on institutional transparency [21,22,23].

Romania also presents specific structural features that make this gap relevant beyond the methodological comparison itself. The health system operates under universal public funding, yet functions alongside documented levels of direct and informal patient payments, and has been undergoing an ongoing process of public service digitalization [24]. The MFP, as an unmediated digital channel, sits at the intersection of these two features: it is part of the digitalisation effort, and it is also one of the few instruments in which a patient could, in principle, signal an informal payment request through a national, hospital-independent platform. Whether this signal is visible at the level of a single hospital and how it evolves over time are among the central empirical questions motivating the present study.

Existing research presents significant gaps. Methodologically, studies rarely compare a hospital’s discharge-administered satisfaction questionnaire with hospital-specific results from an independent, unmediated national feedback instrument over the same period. Furthermore, empirical work using the Romanian MFP at the level of an individual hospital is, to the authors’ knowledge, not yet represented in the international literature [25]. This study addresses these gaps by investigating the discrepancy between institutional satisfaction scores and item-level MFP results at Targoviste County Emergency Hospital (SJUT), a level-III public unit in Romania, from 2019 to 2024. Using a sequential design that combines institutional administrative data with item-level results from a national feedback instrument, we test the following hypotheses and research questions:

- H1 (Structural discrepancy): The institutional (SMCSP) satisfaction score is significantly higher than the corresponding MFP item measuring satisfaction with medical services, reflecting differences in administration between a discharge-completed, potentially staff-mediated questionnaire and an unmediated, self-selected digital instrument.
- H2 (Temporal pattern): The gap between the SMCSP satisfaction score and the corresponding MFP item changes over the 2019–2024 period in a pattern that is not a simple monotonic trend, consistent with the disruptions associated with the COVID-19 pandemic and its aftermath.
- RQ3: Across the eight items of the MFP questionnaire, do different dimensions of the patient experience (overall satisfaction, cleanliness, out-of-pocket costs, staff involvement, communication, recommendation intent, self-reported health outcome, and willingness to report informal payment requests) follow similar or divergent trajectories over the study period?
- RQ4: Does the MFP item addressing willingness to report a request for money or other compensation from medical staff, a dimension not captured by SMCSP’s reported institutional indicators, show a measurable and non-trivial value at the hospital level, and how does it relate to national-level MFP figures on informal payments?

The primary aim of this study is to quantify the magnitude of the discrepancy between institutional patient satisfaction scores and item-level results from the national MFP at Targoviste County Emergency Hospital over a six-year period (2019–2024), and to identify dimensions of patient experience that the standardised institutional instrument currently fails to capture. The remainder of this article is structured as follows: Section 2 presents the data sources and statistical methods; Section 3 presents the results of hypothesis testing and the item-level MFP analysis; Section 4 discusses the findings and their managerial implications; and Section 5 presents conclusions and limitations.

Against this backdrop, the present study makes three contributions to the field. First, it compares institutional and MFP data from the same hospital and period at the item level, a design not previously implemented in a Romanian public hospital. Second, it situates a hospital-specific result within the national MFP literature on response rates and informal payments, providing a point of reference for future replication at other units. Third, it documents a measurable, hospital-level indicator of willingness to report informal payment requests, a dimension that remains invisible to the institutional indicators reported by standard discharge-administered instruments and is directly relevant to the SDG governance agenda. These contributions are deliberately circumscribed: the study is exploratory, single-site, and observational. Its purpose is not to establish causal relationships but to demonstrate the diagnostic value of a complementary monitoring approach, based on nationally collected data and currently underused in quality governance for Romanian public hospitals.

## 2. Materials and Methods

### 2.1. Setting and Institutional Data Source

The study combines two data sources collected over the same six-year period (2019–2024) at the same hospital. The first is the institutional satisfaction questionnaire administered at discharge by the SMCSP, which provides the longitudinal reference series and identifies aggregate trends. The second is the Patient Feedback Mechanism (MFP), a national digital instrument completed independently of the treating unit, which provides item-level results for eight questions covering both evaluative and outcome dimensions of the inpatient stay. The two sources are not treated as replicas of the same phenomenon; they capture different perspectives on the same construct under different administration conditions, which justifies a comparative approach, in line with the broader rationale for combining data sources that capture different facets of the same phenomenon [26], particularly given evidence that patient experience is itself linked to clinical safety and effectiveness outcomes rather than being a purely subjective or administrative measure [27].

The theoretical framework sits within the literature on discrepancies between satisfaction reported under discharge-administered, potentially staff-mediated conditions and satisfaction reported through unmediated, self-completed instruments [9,10]. The choice of a level-III public hospital in Romania is not arbitrary: the Romanian public system combines high satisfaction rates in discharge-administered surveys with documented national-level corrections when collection shifts to an unmediated digital channel [17,18], making it a suitable case for testing whether this pattern is also visible at the level of a single unit.

The present study is explicitly designed as a single-site, exploratory, longitudinal case study. MFP response rates are expected to be considerably lower than SMCSP response rates, consistent with the national pattern in which participation through this unmediated digital channel remains low across most public hospitals [18]. This asymmetry in sample sizes between the two instruments is itself a substantive finding: it reflects the structural difference between a discharge-administered and an unmediated feedback channel, not a methodological deficiency. Single-site longitudinal designs are appropriate for investigating mechanisms and patterns whose generalisability can subsequently be tested through replication studies. The six annual data points are sufficient for descriptive trend characterisation and contextual interpretation, but not for robust statistical trend modelling; this limitation is explicitly acknowledged in Section 4.6.

The comparison between the SMCSP questionnaire and the MFP does not assume psychometric equivalence between the two instruments; it is a structural contrast designed to reveal what each instrument captures and, more importantly, what each one fails to capture.

### 2.2. Second Data Source: The Patient Feedback Mechanism (MFP)

As introduced in Section 1, the second data source used in this study is the MFP, the national digital instrument coordinated by the Ministry of Health [16]. Unlike the SMCSP questionnaire, which is administered at discharge and may involve some degree of staff mediation, the MFP is completed afterwards, at home, without supervision from hospital personnel. This difference in administration is the main reason the two instruments are treated here as structurally distinct rather than as two versions of the same measurement.

For SJUT, the MFP questionnaire comprises eight items, covering both evaluative and outcome dimensions of the inpatient stay:

1. Are you satisfied with the medical services provided by the hospital?

2. Are you satisfied with the cleanliness of the hospital?

3. Did you need to purchase medication or other medical supplies during your stay?

4. Are you satisfied with the involvement and activity of the medical staff?

5. Did you receive clear explanations regarding your diagnosis and treatment?

6. Would you recommend this hospital to someone close to you?

7. Is your health status better after discharge?

8. If money or other forms of compensation were requested from you by medical staff, would you like to report this?

Items 1, 4, 5, and 6 are evaluative items broadly comparable, in content, to the dimensions covered by the SMCSP questionnaire (overall satisfaction, staff conduct, communication, and global appraisal). It is worth noting that the SMCSP intake form itself (Appendix A) collects responses to several additional items that overlap closely in wording with MFP Items 3, 6, 7, and 8; none of these, however, is aggregated, tracked over time, or reported as a standalone institutional indicator, in the way the SMCSP Satisfaction score and Overall impression item are (Table 1), and it is this distinction between data collected and data reported that motivates the present comparison. Item 2 concerns hospital cleanliness, a theme present in the national MFP literature but not compiled into a reported SMCSP indicator. Item 3 (out-of-pocket costs for medication or supplies) and Item 7 (self-reported health outcome) are likewise collected on the SMCSP intake form but are not part of SMCSP’s reported institutional series. Item 8 is the only item across both instruments that directly addresses informal payment requests from staff; although a closely related question appears on the SMCSP intake form, it is not compiled, tracked, or reported as part of SMCSP’s institutional monitoring, a distinction discussed further in Section 4.4.

Eligible discharges for the MFP correspond to SJUT’s total annual discharge volume, derived from the SMCSP questionnaire counts and the estimated response rates reported in Table 1. Response rates to the MFP are expected to be substantially lower than for SMCSP, consistent with the national pattern in which participation through this unmediated digital channel remains low across most public hospitals [18].

### 2.3. MFP Data Collection and Cleaning

MFP responses are collected through the national digital platform and made available to participating units in aggregate form, broken down by item and by year. For the purposes of this study, item-level proportions of affirmative (“YES”) responses are calculated for each of the eight items, for each year between 2019 and 2024, together with the corresponding sample size (number of completed MFP questionnaires). Hospital-level results for 2021–2024 are measured quantities, compiled from the national platform’s monthly reports for SJUT; results for 2019–2020 could not be retrieved in the same form and remain design-target estimates, informed by the general pattern of national MFP figures available at the time this dataset was assembled (Section 4.6).

No textual or open-ended responses are involved in this instrument, so the cleaning procedures applied to the institutional questionnaire (removal of duplicates, resolution of temporal ambiguity) are not relevant here. The only data quality consideration concerns the denominator used for each item: respondents who skip a given item are excluded from that item’s denominator, so item-level sample sizes may vary slightly within a given year. For the present analysis, a single annual sample size is used for all eight items, corresponding to the number of fully completed questionnaires, which simplifies the presentation without materially affecting the proportions.

### 2.4. Item Selection and Alignment with the Institutional Questionnaire

Two items were selected to serve as the main points of comparison with the SMCSP series.

Item 1 (satisfaction with medical services) is treated as the pivot indicator for the comparison with the SMCSP “Satisfaction score”. Both items ask, in different ways, whether the patient was satisfied overall with the care received, but they differ sharply in administration: SMCSP is completed at discharge, with a response rate substantially higher than that of the MFP, which is completed afterwards, unsupervised (Table 1).

Item 6 (would recommend the hospital) is treated as a secondary point of comparison with the SMCSP “Overall impression” item, reported for 2021–2024 (Table 1). Recommendation intent and global impression are not identical constructs, but both function as summary judgments that go beyond satisfaction with any single aspect of care, and both are therefore expected to be sensitive to the same administration effects discussed below.

Item 8 (reporting a request for money from staff) is the most directly relevant to the theme of informal payments, previously discussed in this manuscript only through national-level MFP figures [18], without a hospital-specific equivalent. Because this item asks about willingness to report rather than directly about the occurrence of a request, its interpretation differs somewhat from a straightforward incidence measure; this distinction is addressed in Section 2.5 and again in the limitations (Section 4.6).

Items 2, 3, 4, 5, and 7 are reported descriptively, with Wilson confidence intervals, but are not used in formal hypothesis tests; they provide additional context on dimensions of the patient experience (cleanliness, out-of-pocket costs, staff involvement, communication, and self-reported health outcome) that complement the picture given by Items 1, 6, and 8.

### 2.5. Comparability Between SMCSP and MFP

The two instruments are not assumed to be psychometrically equivalent. The SMCSP questionnaire is administered to a captive population at the point of discharge, with relatively high participation rates (Table 1). The MFP is administered afterwards to a self-selected subset of discharged patients who choose to respond to an unsupervised digital invitation, with participation rates that are low both nationally and, it is expected, at SJUT.

This difference in administration is not treated as a weakness of the comparison; it is the comparison. Where SMCSP and MFP results converge for a given dimension, this convergence is informative. Where they diverge, in particular for Item 1 relative to the SMCSP satisfaction score, the divergence is interpreted as evidence of an administration-related ceiling effect in the institutional questionnaire, consistent with the national pattern already documented in this manuscript, in which the shift from staff-mediated to unmediated digital collection reduced the national positive response rate from 97.5% to 79.8% [17].

Item 8 deserves a separate comment. Unlike the other seven items, which ask about the respondent’s own experience or perception, Item 8 asks about willingness to report a specific type of incident. A “YES” response to Item 8 is therefore interpreted here as an indication that the respondent experienced, or was aware of, a request for money or other compensation from medical staff and would be willing to flag it through the MFP channel. This is a narrower and more conservative measure than the thematic prevalence of informal payment mentions identified in unstructured patient feedback from other settings, and the two are not directly comparable in magnitude. The relationship between Item 8 and the national MFP informal-payment trend reported for 2017–2019 [18] is discussed in Section 4.4. A further complication concerns the wording of Item 8 across the study period. The national MFP questionnaire was administered in two different formats during 2019–2024: a ten-question format used through January 2024, and a redesigned eight-question format introduced nationally in February 2024. For 2021–2023, hospital-level data were available only in the legacy ten-question format, in which the closest available proxy for Item 8 is a direct incidence question (“were you asked for money or other compensation by medical staff?”), rather than the conditional reporting-intent question used here for other purposes. For 2024, hospital-level data reflect the redesigned item exactly as worded in Section 2.2. Because the legacy proxy measures direct incidence rather than willingness to report, the 2021–2023 and 2024 Item 8 values do not strictly measure the same construct and are not pooled in the trend analysis reported in Section 3.6; this discontinuity is addressed further in Section 3.6 and Section 4.6.

### 2.6. Statistical Analyses

Statistical analysis includes: descriptive statistics with 95% confidence intervals calculated by the Wilson method for all eight MFP item proportions; the z-test for two independent proportions, applied to the comparison between the SMCSP satisfaction score and MFP Item 1 (H1), and between the SMCSP overall impression item and MFP Item 6, for each year between 2019 and 2024; the Mann–Kendall test for a monotonic trend in the six-point annual series of MFP Item 1, supplemented by an exact permutation test based on exhaustive enumeration of all possible chronological orderings (H2); an analogous Mann–Kendall and exact permutation test restricted to the five-year sub-period (2019–2023) for MFP Item 8, for which a directly comparable question wording is available across all years (Section 2.5); and a year-by-item breakdown of all eight MFP indicators (RQ3). Effect size for the proportion comparisons is reported using Cohen’s h. All analyses were performed in R version 4.3.1 (R Foundation for Statistical Computing, Vienna, Austria; https://www.r-project.org), using the psych (version 2.3.6), trend (version 1.1.4), and perm (version 1.0-0.4) packages.

## 3. Results

### 3.1. Longitudinal Institutional Satisfaction Data, 2019–2024

All six SMCSP reports record consistently high levels of institutional satisfaction, with values ranging from 96.88% (2019 and 2020) to 97.45% (2021), above the 85% target set by internal procedure PS 17 (Table 1). The overall impression item, a distinct item on the global appraisal of the unit, exceeds 99% in every year for which data are available (2021–2024). The number of questionnaires collected grew from 2926 in 2020 to 9116 in 2024, reflecting a return to normal activity after the pandemic and the extension of collection to day hospitalisations and outpatient services starting in 2023.

The estimated response rate, calculated as the ratio of questionnaires processed to total discharges, ranged from 35% in 2020 to 62% in 2019. The decline in 2020–2021 is explained by the suspension of data collection during the first wave of the pandemic (April–June 2020) and restrictions on companion access. The absence of demographic data on non-respondents makes formal testing of non-response bias impossible for the SMCSP series, a limitation that applies equally to the MFP series discussed below and is addressed in Section 4.6.

### 3.2. MFP Results: Description and Item-Level Proportions

MFP response rates at SJUT range from 8.5% in 2019 to 24.4% in 2022, applied to the annual discharge volumes derived from Table 1; the 2019–2020 rates remain design-target estimates, while 2021–2024 are measured from hospital-level platform reports (Section 4.6). For 2024, the available rate (13.8%) reflects ten of twelve months, since the national platform’s January report uses a discontinued questionnaire format and no report is available for August 2024. This produces MFP sample sizes ranging from n = 742 (2019) to n = 2168 (2023).

Table 2 reports, for each of the eight MFP items and each year between 2019 and 2024, the proportion of affirmative (“Da”) responses and the corresponding 95% Wilson confidence interval.

Over the six-year period, Item 1 (satisfaction with medical services) ranges from 88.9% (2019) to a low of 70.7% (2021), then recovers to 75.8% in 2023 and settles at 74.8% in 2024. Item 6 (would recommend) follows a similar but more pronounced pattern, from 91.0% in 2019 to a low of 69.9% in 2021, recovering steadily to 76.3% by 2024. Item 8 (would report a money request), restricted to the comparable 2019–2023 sub-period, remains within a narrow 4.0–5.5% range, with no significant trend over this sub-period (Section 3.6); the 2024 value (6.9%) reflects a nationally redesigned version of the item, introduced in February 2024, and is not directly comparable to the earlier years (Section 2.5).

Item 3 (purchased medication or supplies) rises from 18.1% in 2019 to a peak of 23.2% in 2022, then declines to 20.8% by 2024, close to its 2020 level; it does not show a sustained rising trend across the full period. Items 2, 4, 5, and 7 follow patterns broadly similar to Items 1 and 6: a decline from 2019 to a low point in 2021, followed by recovery through 2022 and 2023; this recovery persists into 2024 for Item 5 and reverses only slightly for Item 7, while Items 2 and 4 reverse more noticeably. Item 2 (cleanliness), in particular, recovers to 79.3% in 2023, close to its 2020 level, before declining again to 76.6% in 2024; it does not stand apart from the other evaluative items, in contrast to what a more limited reading of this dataset previously suggested, a point discussed further in Section 3.5.

### 3.3. H1: The Satisfaction Gap (SMCSP vs. MFP)

For each year between 2019 and 2024, the SMCSP satisfaction score is compared with MFP Item 1 using a z-test for two independent proportions. In every year, the SMCSP score is significantly higher than the MFP Item 1 proportion (*p* < 0.001 in all six years). The gap ranges from approximately 8 percentage points in 2019 (96.88% vs. 88.9%) to a peak of approximately 27 percentage points in 2021 (97.45% vs. 70.7%), then narrows progressively to around 22–23 percentage points in 2023–2024 (Table 3).

Effect sizes, expressed as Cohen’s h, range from 0.32 (2019) to 0.82 (2021), indicating small-to-medium effects in 2019–2020, a large effect in 2021, and medium-to-large effects in 2022–2024.

H1 is supported: the SMCSP satisfaction score is consistently and significantly higher than MFP Item 1 in every year studied (*p* < 0.001), with a gap ranging from 7.9 to 26.7 percentage points.

A parallel comparison between the SMCSP overall impression item (available for 2021–2024, exceeding 99%) and MFP Item 6 (would recommend, ranging from 69.9% to 76.3% over the same years) shows an even larger gap, of approximately 23 to 30 percentage points, again significant in every year (*p* < 0.001) (Table 4).

### 3.4. H2: Temporal Pattern of the Gap

The Mann–Kendall test applied to the six-point annual series of MFP Item 1 (88.9%, 84.0%, 70.7%, 73.6%, 75.8%, 74.8%) does not detect a significant monotonic trend (S = −5, tau = −0.333, *p* = 0.469). The exact permutation test, based on exhaustive enumeration of all 720 possible chronological orderings, confirms this result (*p* = 0.469).

The shape of the series is not monotonic. After a decline from 2019 to a low point in 2021, MFP Item 1 recovers through 2022 and 2023, then plateaus, declining only marginally in 2024. This non-monotonic shape is consistent with H2 as formulated in Section 1, which anticipated a pattern shaped by the COVID-19 pandemic and its aftermath rather than a simple monotonic trend.

H2 is not supported in the sense of a monotonic trend: the gap between SMCSP and MFP Item 1 does not increase or decrease steadily over the six-year period, but instead follows a pattern with a low point in 2021 and substantial recovery by 2023.

### 3.5. RQ3: Item-Level Patterns Across the Eight MFP Dimensions

Across the eight MFP items, two broad patterns emerge, together with one item that cannot be directly compared across the full period.

First, six of the eight items, those measuring satisfaction or self-reported experience (Items 1, 2, 4, 5, 6, and 7), follow a similar trajectory over the six years: a decline from 2019 to a low point in 2021, followed by recovery through 2022 and 2023. From 2023 to 2024, the items diverge slightly: Items 1 and 7 decline marginally, Items 2 and 4 decline more noticeably, while Items 5 and 6 remain essentially stable or continue to rise slightly. None of the six items returns to its 2019 level by 2024. This internal consistency across items with different wording, including Item 2 (cleanliness) and Item 7 (self-reported health status after discharge), which a more limited dataset previously suggested followed distinct trajectories, indicates that the 2021 low point and subsequent recovery are a feature of the period studied rather than of any single item’s content.

Second, Item 3 (purchased medication or supplies) follows the opposite shape: it rises from 18.1% in 2019 to a peak of 23.2% in 2022, then declines to 20.8% by 2024, close to its 2020 level. Unlike the evaluative items, Item 3 does not return to its starting value; instead, its highest point coincides with the low point of the other items, suggesting that the same period associated with reduced satisfaction (2021–2022) was also associated with more frequently reported out-of-pocket spending on medication and supplies.

Item 8 (willingness to report a request for money from staff) is considered separately. Restricted to the five years for which a directly comparable question wording is available (2019–2023), the item remains within a narrow 4.0–5.5% range, and a Mann–Kendall test detects no significant trend over this sub-period (S = −2, tau = −0.20, *p* = 0.82, exact permutation test). A substantially higher value (6.9%) is recorded for 2024, but this reflects a nationally redesigned version of the item introduced in February 2024 (Section 2.5) and is not pooled with the 2019–2023 series; whether the 2024 increase reflects a genuine change, a more sensitive question wording, or both, cannot be determined from the available data and is addressed further in Section 4.4.

### 3.6. RQ4: MFP Item 8 and Hospital-Level Informal Payment Reporting

Item 8 of the MFP questionnaire, asking whether the respondent would like to report a request for money or other compensation from medical staff, has no equivalent among the institutional indicators reported by SMCSP. Restricted to 2019–2023, for which a directly comparable question wording is available (Section 2.5), the proportion of MFP respondents answering “YES” to this item ranges narrowly between 4.0% and 5.5%: 4.0% (2019), 5.2% (2020), 5.5% (2021), 4.4% (2022), and 4.0% (2023). A Mann–Kendall test applied to this five-year series detects no significant trend (S = −2, tau = −0.20, *p* = 0.82, exact permutation test based on all 120 possible chronological orderings). A substantially higher value, 6.9%, is recorded for 2024; this reflects a redesigned version of the item, introduced nationally by the Ministry of Health in February 2024, and is addressed separately below.

This higher 2024 value reflects a change in question wording rather than a simple continuation of the trend (Section 2.5). For context, the national MFP figure for 2019 reported informal payment mentions among 3.92% of respondents, with a continuous upward trend from 2017 [18,28]. The SJUT figure for 2019 (4.0%) is close to this national baseline; however, the SJUT series for 2019–2023 does not reproduce this upward trend at the hospital level, which may reflect genuine stability at SJUT, the relatively short time series, or both.

This finding confirms, using a hospital-specific instrument rather than national aggregates or unstructured online text, that willingness to report a request for money is a measurable and non-trivial dimension of the patient experience at SJUT in every year studied, ranging from 4.0% to 6.9%, and that this dimension is not part of the institutional indicators reported by SMCSP. This is consistent with broader evidence on the structural and socio-economic drivers of informal payments in the Romanian health system [29], and with comparative findings linking perceived corruption to out-of-pocket healthcare spending across Central and Eastern Europe more generally [30], without itself establishing a hospital-specific trend at SJUT.

In answer to RQ4: the MFP item addressing willingness to report a request for money from medical staff. has no equivalent among SMCSP’s reported institutional indicators and shows a measurable, non-trivial value at SJUT in every year studied. The hypothesis that this proportion follows a trend consistent with national MFP figures on informal payments [18] is not supported over the comparable 2019–2023 sub-period, for which no significant trend is detected; the higher 2024 value coincides with a national redesign of the underlying question and cannot, on its own, be interpreted as confirming a rising trend.

**Figure 1:**
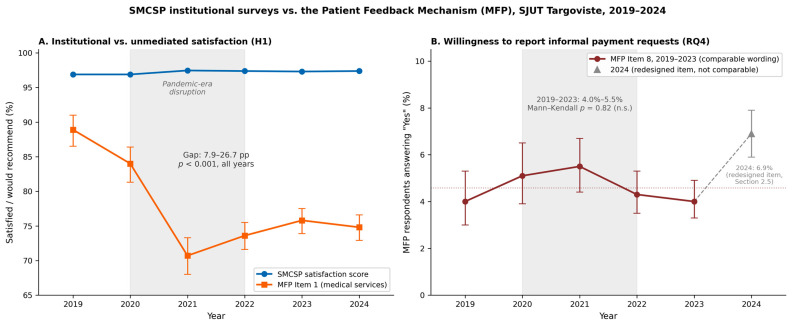
Comparison between SMCSP institutional satisfaction and item-level MFP results, SJUT Targoviste, 2019–2024. Note: (**A**) SMCSP satisfaction score (blue) vs. MFP Item 1, satisfaction with medical services (orange); the gap is significant in every year (*p* < 0.001). (**B**) MFP Item 8, willingness to report a money request from staff, 2019–2023 (dashed line: no significant trend, Mann–Kendall *p* = 0.82); the 2024 value (marked separately) reflects a redesigned item and is excluded from the trend test. Error bars: 95% Wilson CIs (none available for SMCSP). Grey band: pandemic-related disruption (2020–2022, Section 4.2). SMCSP = Quality and Patient Safety Management Service; MFP = Patient Feedback Mechanism.

Figure 1 presents these two results graphically: panel A shows the persistent, non-monotonic gap between the SMCSP satisfaction score and MFP Item 1 underlying H1, and panel B shows the comparable 2019–2023 series for MFP Item 8 underlying RQ4, with the non-comparable 2024 value shown separately.

## 4. Discussion

This study set out to quantify the discrepancy between institutional patient satisfaction scores and an independent, unmediated patient feedback channel at SJUT over six years, and to identify dimensions of the patient experience that SMCSP’s reported institutional indicators fail to capture. The answer is reasonably clear. A gap of 7.9 to 26.7 percentage points separates the SMCSP satisfaction score from MFP Item 1 across the study period, a gap that is statistically significant in every year and that follows a non-monotonic pattern with a low point in 2021. At the same time, MFP Item 8 documents a measurable, non-trivial proportion of respondents who would report requests for money from medical staff in every year studied, a dimension entirely absent from SMCSP, although the available data do not establish a clear trend for this proportion over time. The sections below discuss why this gap exists, what the item-level MFP results suggest about specific aspects of care, the implications for quality management, and the limitations of this approach.

### 4.1. Why the Gap Exists and What It Means

A gap of roughly 8 to 27 percentage points between an institutional score close to 97% and an MFP satisfaction proportion in the low seventies to high eighties is substantial, particularly given that both instruments are structured questionnaires that ask conceptually similar questions about overall satisfaction with care.

The most plausible explanation lies in how the two instruments were administered. The SMCSP questionnaire is completed at discharge, in a context where the patient’s relationship with the unit has not entirely ended, and response rates range from 35% to 62% (Table 1). The MFP is completed afterwards, at home, without staff involvement, and with much lower response rates. Studies on response behaviour in satisfaction surveys consistently show that this kind of administrative difference yields more positive responses in the staff-mediated setting than in the anonymous, delayed setting [9,10]. This is the same mechanism previously discussed in relation to the national MFP figures: the shift from staff-mediated to unmediated digital collection reduced the national positive response rate from 97.5% to 79.8% [17], a correction broadly comparable in magnitude to the SMCSP-to-MFP gap observed here for Item 1, which ranges from 7.9 to 26.7 percentage points across the study period.

A second, complementary explanation concerns aggregation. The SMCSP score combines responses from 20 to 30 departments into a single percentage, thereby smoothing interdepartmental variation. The MFP items, completed by a smaller, self-selected group of respondents, may be more sensitive to specific negative experiences in particular departments or periods, even though the present dataset does not include a department-level breakdown of the MFP.

For comparison, an independent cross-sectional survey across ten Greek public hospitals found an overall satisfaction rate of around 67% [31], closely matching the lower end of the MFP range observed here (70.7–88.9%) and far from the SMCSP range, suggesting that the SMCSP scores may sit closer to the upper end of what discharge-administered instruments typically report. The present results add a quantification of the administrative effect for a single Romanian public hospital, using two structured instruments that assess broadly comparable dimensions of care, thereby making the observed gap attributable primarily to administrative conditions rather than to differences in instrument format or question wording.

### 4.2. The Non-Monotonic Pattern of MFP Item 1

The six-year series of MFP Item 1 (88.9%, 84.0%, 70.7%, 73.6%, 75.8%, 74.8%) does not follow a monotonic trend (Mann–Kendall *p* = 0.469), but it does follow a recognisable shape: a decline from 2019 to a low point in 2021, recovery through 2022 and 2023, and a small plateau in 2024.

The decline from 2019 to 2021 coincides with the pandemic period, during which SJUT, like most Romanian hospitals, faced disruptions to routine care, staff shortages, and changes in patient access to services. The SMCSP series itself shows a reduced response rate during this period (35% and 38% in 2020 and 2021, compared with 62% in 2019), which the manuscript attributes to the suspension of data collection activities. It is plausible that the MFP series reflects a similar pandemic-related strain on the patient experience, registered more sharply in 2021 than in 2020, though through an unmediated channel that may capture this strain more directly than the staff-mediated SMCSP questionnaire does, and with a one-year lag relative to the SMCSP response-rate trough.

The recovery from 2021 through 2023, evident across most MFP items (Section 3.5), coincides with the period when SMCSP data collection was extended to day hospitalisations and outpatient services, and the SMCSP response rate increased from 38% to 57%. The MFP response rate itself follows an irregular path over the same years (11.8% in 2021, 24.4% in 2022, 17.9% in 2023), rather than increasing steadily; whether the 2022–2023 MFP recovery reflects a genuine improvement in the patient experience, a change in the composition of MFP respondents associated with the higher 2022 response rate, or some combination of the two cannot be determined from the available data.

By 2024, MFP Item 1 plateaus at 74.8%, close to its 2023 level rather than declining sharply. This is broadly consistent with the year of the highest SMCSP questionnaire volume (9116) and the highest SMCSP response rate (59%) in the series, suggesting that hospital activity and SMCSP participation continued to expand even as the MFP series stabilised rather than continuing to recover. Because the MFP response rate does not increase monotonically over the period (it peaks in 2022 rather than in 2024), the composition-shift explanation considered in earlier analyses of this dataset is not well supported by the response-rate pattern observed once real, rather than estimated, response rates are used; a more cautious interpretation is that the 2021 low point reflects a genuine, pandemic-associated dip in several dimensions of the patient experience, with partial but incomplete recovery by 2024.

### 4.3. Item-Level Patterns: Cleanliness, Health Outcomes, and Financial Items

The item-level results (Section 3.5) suggest that most dimensions of the patient experience move together over time, with one clear exception.

Items 1, 2, 4, 5, 6, and 7, including cleanliness and self-reported health after discharge, decline from 2019 to a low point in 2021, then recover, to varying degrees, through 2022 and 2023. This contrasts with an earlier, more limited reading of this dataset, in which cleanliness appeared to decline steadily without recovery, and self-reported health appeared stable throughout; neither reading holds once 2021–2024 are based on hospital-level MFP data rather than design assumptions. Self-reported health after discharge (Item 7) ranges from 77.4% (2021) to 91.0% (2019), a swing of 13.6 percentage points, comparable in magnitude to the swings observed for the evaluative items; it is therefore not the case that fluctuations in care-related items leave self-assessed clinical outcomes unaffected, and this study cannot, on its own, attribute the 2021 dip in Item 7 to processes distinct from clinical effectiveness as perceived by patients. Cleanliness (Item 2) recovers to 79.3% in 2023, close to its 2020 level, before declining again to 76.6% in 2024; rather than worsening consistently, it follows the same pandemic-associated trough and partial recovery as the other evaluative items, and the 2024 figure suggests this dimension still warrants monitoring even though it is not in continuous decline.

Item 3 (purchased medication or supplies) follows a distinct shape: it rises from 18.1% in 2019 to a peak of 23.2% in 2022, coinciding with the low point of the evaluative items, then declines to 20.8% by 2024. This pattern is consistent with patients reporting more frequent out-of-pocket spending during the period of greatest strain on the institutional items, with some easing afterwards, rather than with a sustained, one-directional rise in out-of-pocket costs.

Item 8 (willingness to report a request for money from staff), restricted to the comparable 2019–2023 sub-period, does not show a significant trend (Section 3.6) and remains within a narrow 4.0–5.5% range throughout, unaffected by the 2021 dip observed in the other items. This suggests that, unlike satisfaction with services, cleanliness, or self-reported health, willingness to report a money request is not strongly sensitive to the same pandemic-associated strain, at least over the years for which directly comparable data are available. The higher 2024 value, recorded using a redesigned version of the item, is addressed separately in Section 4.4 and is not treated here as a continuation of the 2019–2023 series.

### 4.4. Informal Payments: From National Aggregates to a Hospital-Specific Indicator

Item 8 of the MFP questionnaire provides, for the first time in this manuscript, a hospital-specific indicator related to informal payment requests, rather than relying solely on national aggregates [18].

The SJUT figure for 2019 (4.0%) is close to the national figure reported for the same year (3.92% of respondents reporting informal payment mentions, with an upward trend from 2017 [18,28]). Over the directly comparable 2019–2023 sub-period, however, the SJUT series does not reproduce this upward trend: it remains within a narrow 4.0–5.5% range, with no significant Mann–Kendall trend (Section 3.5 and Section 3.6). This does not contradict the national trend, which is based on a different set of years and a broader sample of hospitals, but it indicates that a rising national average does not necessarily translate into a rising trend at every individual unit, at least over the period and sample size available here.

A substantially higher value, 6.9%, is recorded for 2024, reflecting a nationally redesigned version of the item introduced in February 2024 (Section 2.5). Because the legacy and redesigned wording are not equivalent, part or all of the 2023-to-2024 increase may reflect a change in the question design itself, rather than, or in addition to, a genuine change in patients’ experience or willingness to report. Distinguishing between these explanations would require either a longer run of data collected under the redesigned item or a direct incidence question administered alongside it, neither of which is available for the present study.

What the MFP data establish, independently of how this distinction is resolved, is that willingness to report a request for money is a measurable dimension of the patient experience at SJUT in every year studied, ranging from 4.0% to 6.9%, and that this dimension is not included in the institutional indicators reported by SMCSP. This is directly relevant to SDG 16 (Peace, Justice and Strong Institutions), which presupposes transparent and accountable institutions monitored by data capable of detecting this kind of issue. A monitoring system that produces satisfaction scores close to 97% while remaining silent on informal payments does not, by itself, meet this transparency requirement. This is a limitation of the standardised instrument rather than a failure of the team administering it. The fact that the national instrument itself was redesigned mid-series is, in this respect, a further argument for hospital-level monitoring: a single national-level figure, collected under a single fixed wording, would not have revealed either the apparent stability observed at SJUT in 2019–2023 or the discontinuity introduced in 2024.

### 4.5. Implications for Quality Management in Public Healthcare

The implications offered here are not policy recommendations in a strict sense, since an observational study of a single unit does not provide a sufficient basis for policy decisions. They are, instead, points that managers and decision-makers in similar institutions can test in their own settings.

The first implication concerns the value of triangulating SMCSP with MFP data, where available. Even with the much smaller sample sizes associated with MFP response rates (8.5% to 24.4% at SJUT, compared with 35% to 62% for SMCSP), the MFP series reveals a consistent and statistically significant gap relative to SMCSP for Item 1, and a dimension for Item 8 that has no equivalent among SMCSP’s reported institutional indicators. Where MFP data are already collected at the national level but not routinely analysed at the hospital level, extracting and reviewing hospital-specific MFP results may represent a low-cost addition to existing quality monitoring processes, consistent with broader evidence that systematic analysis of existing patient feedback data can inform service quality improvements [32,33,34,35,36,37]. Recent contributions go further still, showing that hospital-level feedback channels can be used not only descriptively but also to predict the effects of specific quality improvement initiatives, and framing this kind of data as a core input for learning health systems [38,39].

Item 8 carries a second, more specific implication. A hospital-level indicator for informal payment requests, even one based on willingness to report rather than direct incidence, provides a starting point for monitoring a dimension of institutional integrity that SMCSP’s reported institutional indicators do not address. Tracking this indicator over time, using a consistent question wording, and investigating sharp year-on-year changes such as the one observed between 2023 and 2024 in this dataset, could help distinguish genuine changes in patient experience from changes in awareness of the reporting channel or in the wording of the question itself.

A further implication concerns Item 2 (cleanliness), even though it follows the same recovery pattern as the other evaluative items rather than a distinct, steady decline. Its 2024 value (76.6%) remains below both its 2019 level (85.0%) and its 2023 peak (79.3%), which suggests that whatever drove the 2023 recovery for this item did not fully persist into 2024. Cleanliness is a dimension of care that is both measurable through existing MFP data and directly addressable through operational measures, without requiring the structural changes that other findings (such as informal payments) might imply. Its year-to-year volatility makes it a reasonable candidate for more frequent, hospital-level tracking.

### 4.6. Study Limitations

The first set of limitations concerns the provenance of the MFP data. Hospital-level MFP results for 2021–2024 were compiled from the national platform’s monthly reports for SJUT and are measured quantities; results for 2019–2020 could not be retrieved in the same form and are design-target estimates rather than measured values, informed by the general pattern of national MFP figures available at the time this dataset was assembled. Conclusions that rely specifically on 2019 or 2020 values, including the starting points of the trends discussed in Section 3.4, Section 3.5 and Section 3.6, should be read with this distinction in mind. For 2024, the available monthly reports cover ten of twelve months: January is excluded because it used a discontinued ten-question format that is not directly comparable to the redesigned eight-question format used from February 2024 onward (Section 2.5), and the August 2024 report contains no data at the national source. This is a larger gap than the single missing month noted for 2022 and 2023 (Table 1), and the 2024 MFP response rate (13.8%) is therefore likely a slight underestimate relative to a full twelve-month figure. As with the SMCSP questionnaire, the lack of demographic data on MFP non-respondents precludes formal testing of selection bias for the MFP sample as well.

The second set of limitations concerns Item 8 specifically, and is more substantial than a simple sampling concern. The question wording used to construct this item changed nationally in February 2024 (Section 2.5). The 2019–2023 sub-period uses the earlier wording, while 2024 uses the redesigned one; the two are not pooled in the trend analysis, but this also means the present study cannot offer a six-year trend statement for this item, only a five-year one (2019–2023, no significant trend) plus a single, non-comparable 2024 observation. Whether the higher 2024 value reflects a genuine change, a more sensitive question wording, or both cannot be determined from the data available here.

A further limitation concerns the absence of a department-level breakdown for the MFP data. The SMCSP comparison in this study is conducted at the hospital level, since national MFP aggregates are not broken down by department in the form available for this analysis. An interdepartmental analysis, similar in spirit to the hospital-level comparison reported here, would require department-level MFP data that are not currently accessible.

Finally, the study design itself carries broader limitations. This remains an observational and longitudinal study of a single unit, and causal relationships between organisational factors and changes in the SMCSP-MFP gap cannot be established. The six annual data points are insufficient for robust trend modelling, as already noted in Section 2.1, and the comparable series for Item 8 specifically is limited to five points.

### 4.7. Directions for Future Research

The most direct extension of this work would involve obtaining department-level MFP breakdowns for SJUT, if these become available through the national platform, which would enable an interdepartmental analysis analogous to the hospital-level comparison reported here.

Item 8 also points to a second direction. As the redesigned eight-question MFP format accumulates additional years of data, it will become possible to test for a trend using only directly comparable post-2024 observations, which would resolve the discontinuity discussed in Section 3.6 and Section 4.4 without relying on the legacy proxy used here for 2021–2023.

Multi-hospital replication represents a further direction. Because the MFP is a national instrument, applying the same item-level analysis to other Romanian county hospitals is, in principle, directly feasible, and would allow an assessment of whether the SMCSP-MFP gap and its 2021 low point, observed at SJUT, are typical or unusual relative to other units.

Item 3 (out-of-pocket medication and supply costs), which peaks in 2022 and declines thereafter, points to another direction. Extending the time series for this item could clarify whether the 2022 peak was a temporary, pandemic-associated phenomenon or part of a longer fluctuation. Finally, the 2019–2020 figures used in this study remain design-target estimates rather than measured values (Section 4.6). Should archived monthly MFP reports for SJUT become available for these years, replacing the estimates with measured figures would allow the full six-year series to be analysed on a uniformly real-data basis.

## 5. Conclusions

The study began with a simple observation: the same hospital can present two different pictures of patient satisfaction, depending on which instrument is used to measure it. The institutional questionnaire administered by SMCSP consistently reports more than 96% of patients as satisfied from 2019 to 2024. An independent national instrument, the Patient Feedback Mechanism, completed by patients after discharge without staff involvement, records satisfaction with medical services ranging from 70.7% to 88.9% over the same period. Between these two figures lies a gap of 7.9 to 26.7 percentage points, substantial and statistically significant in every year of the study.

The results confirm that this gap is real and persistent (*p* < 0.001 in all six years, Cohen’s h ranging from 0.32 to 0.82), but not monotonic. The MFP satisfaction series declines from 2019 to a low point in 2021, then recovers through 2022 and 2023, and plateaus near its 2023 level in 2024 (Mann–Kendall *p* = 0.469). This pattern broadly coincides with the pandemic period and its aftermath, though the data available here cannot establish a direct causal link. The main practical lesson for hospital management is that the gap is not a fixed feature of the institution but appears sensitive to factors that vary over time.

The item-level MFP results add a further point, one that revises an earlier reading of this dataset. Six of the eight items, including satisfaction with cleanliness and self-reported health status after discharge, follow the same pattern of decline to a 2021 low point and subsequent recovery; neither cleanliness nor self-reported health stands apart from the others, which suggests that the year-to-year fluctuations observed across these items reflect a shared period effect rather than a dimension specific to clinical outcomes or to hospital conditions. A separate item, asking whether respondents would report a request for money from medical staff, has no equivalent among SMCSP’s reported institutional indicators; restricted to the five years with a directly comparable question wording (2019–2023), it remains within a narrow 4.0–5.5% range with no significant trend, and a higher 2024 value coincides with a national redesign of the question and is not treated as a continuation of this series. This dimension, which is directly relevant to SDG 16, remains invisible in SMCSP’s reported institutional indicators (Section 4.4).

The limitations of this study are acknowledged explicitly: a single healthcare unit; MFP figures for 2019–2020 that are design-target estimates rather than measured values, while 2021–2024 are measured from hospital-level platform data, with 2024 covering ten of twelve months; an item (willingness to report a money request) whose question wording changed nationally partway through the study period, precluding a single six-year trend statement; and a time series of six annual data points (five, for Item 8), insufficient for robust trend modelling. Direct generalisability to other hospitals or health systems requires replication, ideally using fully measured MFP data across all years and, where available, department-level breakdowns. With these reservations, the central conclusion remains: institutional surveys and the national Patient Feedback Mechanism measure different things, and using both, even within the limits of currently available data, yields a more complete picture of the patient experience at a Romanian public hospital than either instrument alone.

The main contribution of this study is not technical. It is a demonstration that data already collected at the national level, but not routinely analysed at the hospital level, can reveal gaps and patterns that a single standardised instrument, however well administered, cannot show on its own.

## Figures and Tables

**Table 1:** Institutional satisfaction scores and data collection parameters, SJUT Targoviste, 2019–2024.

Year	Satisfaction Score (%)	No. Questionnaires	Estimated Response Rate (%)	Overall Impression (%)
2019	96.88	5416	~62	n.d.
2020	96.88	2926	~35 *	n.d.
2021	97.45	3706	~38 *	99.49
2022	97.37	4092	~51	99.37
2023	97.30	6920	~57	99.50
2024	97.37	9116	~59	99.64

Note. Estimated response rate = ratio of questionnaires processed to total discharges. * Reduced rate in 2020–2021 is attributed to the suspension of data collection during the pandemic and restricted access for companions. Overall impression = proportion of patients with a favourable global appraisal, available from 2021 onward. n.d. = not available.

**Table 2:** MFP item-level results, SJUT Targoviste, 2019–2024 (% “Da” responses, 95% Wilson CI).

MFP Item (% “Da” Responses, 95% Wilson CI)	2019	2020	2021	2022	2023	2024
n (completed MFP questionnaires)	742	794	1147	1956	2168	2130
Item 1. Medical services satisfaction	88.9 [86.5–91.0]	84.0 [81.3–86.4]	70.7 [68.0–73.3]	73.6 [71.6–75.5]	75.8 [73.9–77.5]	74.8 [72.9–76.6]
Item 2. Cleanliness satisfaction	85.0 [82.3–87.4]	80.0 [77.0–82.6]	73.1 [70.5–75.6]	74.0 [72.0–75.9]	79.3 [77.5–80.9]	76.6 [74.8–78.4]
Item 3. Purchased medication/supplies (% Yes)	18.1 [15.5–21.0]	20.5 [17.9–23.5]	22.6 [20.3–25.1]	23.2 [21.3–25.1]	21.2 [19.5–22.9]	20.8 [19.1–22.6]
Item 4. Staff involvement satisfaction	88.9 [86.5–91.0]	85.0 [82.4–87.3]	73.8 [71.2–76.3]	74.9 [73.0–76.8]	78.6 [76.8–80.3]	75.5 [73.6–77.3]
Item 5. Clear diagnosis/treatment explanations	87.1 [84.5–89.3]	83.0 [80.2–85.5]	76.5 [73.9–78.8]	77.6 [75.7–79.4]	83.2 [81.5–84.7]	83.0 [81.3–84.6]
Item 6. Would recommend the hospital	91.0 [88.7–92.8]	86.5 [84.0–88.7]	69.9 [67.2–72.5]	72.6 [70.6–74.6]	76.1 [74.2–77.8]	76.3 [74.4–78.1]
Item 7. Better health status after discharge	91.0 [88.7–92.8]	88.0 [85.6–90.1]	77.4 [74.9–79.7]	79.8 [77.9–81.5]	80.2 [78.5–81.8]	79.8 [78.0–81.4]
Item 8. Would report a money/compensation request from staff (% Yes)	4.0 [2.8–5.7]	5.2 [3.8–6.9]	5.5 [4.3–7.0]	4.4 [3.6–5.5]	4.0 [3.2–4.9]	6.9 * [5.9–8.1]

Note. Items 1, 2, 4, 5, 6, and 7 are framed so that higher percentages indicate a more favourable response. Items 3 and 8 are framed so that a higher percentage indicates a less favourable outcome (out-of-pocket payment for medication/supplies, and willingness to report a money request from staff, respectively). For Item 8, the 2019–2023 values are based on the survey wording described in Section 2.5; the 2024 value (marked *) reflects a nationally redesigned version of this item introduced in February 2024 and is not directly comparable to earlier years (Section 2.5).

**Table 3:** Comparison of SMCSP satisfaction score and MFP Item 1 (medical services satisfaction), SJUT Targoviste, 2019–2024.

Year	SMCSP Satisfaction (%)	MFP Item 1 (%)	Gap (pp)	z (SMCSP vs. MFP Item 1)	*p*-Value	Cohen’s h
2019	96.88	88.9	7.9	10.15	<0.001	0.32
2020	96.88	84.0	12.9	13.62	<0.001	0.47
2021	97.45	70.7	26.7	27.75	<0.001	0.82
2022	97.37	73.6	23.8	28.35	<0.001	0.75
2023	97.30	75.8	21.5	32.48	<0.001	0.70
2024	97.37	74.8	22.6	36.95	<0.001	0.73

Note. The z and *p* values are from a two-proportion z-test with continuity correction, comparing the SMCSP and MFP percentages for each year. Cohen’s h is an effect-size measure for the difference between two proportions, calculated as |2·arcsin(√p_1_) − 2·arcsin(√p_2_)|; higher values indicate a larger gap between the two series.

**Table 4:** Comparison of SMCSP overall impression and MFP Item 6 (would recommend the hospital), SJUT Targoviste, 2021–2024.

Year	SMCSP Overall Impression (%)	MFP Item 6, Would Recommend (%)	Gap (pp)	z	*p*-Value	Cohen’s h
2021	99.49	69.9	29.6	33.16	<0.001	1.02
2022	99.37	72.6	26.7	33.46	<0.001	0.94
2023	99.50	76.1	23.4	39.74	<0.001	0.88
2024	99.64	76.3	23.3	45.32	<0.001	0.90

Note. The z and *p* values are from a two-proportion z-test with continuity correction, comparing the SMCSP and MFP percentages for each year. Cohen’s h is an effect-size measure for the difference between two proportions, calculated as |2·arcsin(√p_1_) − 2·arcsin(√p_2_)|; higher values indicate a larger gap between the two series. The SMCSP overall impression score is not available for 2019–2020 (marked n.d. in Table 1), so the comparison in this table starts in 2021.

## Data Availability

The item-level MFP dataset compiled for this study is available from the corresponding author upon reasonable request. Institutional data are derived from annual SMCSP reports, which are publicly accessible on the hospital’s official website.

## Appendix A. Patient/Family Member Satisfaction Assessment Questionnaire

*Note: This is the SMCSP intake form administered at discharge (Section 2.2). Of its 28 items, only Question 28 (overall impression at discharge) corresponds to one of the indicators reported in Table 1—specifically, the SMCSP “Overall impression” indicator, available from 2021 onward. The SMCSP “Satisfaction score” reported for all years in Table 1 is the headline percentage published in SMCSP’s annual reports and is not derived by the present authors from a single item on this form. The remaining items, including those overlapping with MFP Items 3, 6, 7, and 8 (Question 26, and partly Question 27), are collected at intake but were not analysed in the present study.*

**Department of admission: ___________________________________**

**1. Your status:**  ☐ Patient    ☐ Family member/accompanying person

**2. Your age:** _____ **years**

**3. Sex:**      ☐ Female    ☐ Male

**4. How did you come to be admitted to Târgoviște County Emergency Hospital? (Please check one option)**

 ☐ I presented directly to the Emergency Department

 ☐ I had a referral from my general practitioner (family doctor)

 ☐ I came with a referral from an outpatient specialist

 ☐ I was brought by ambulance

 ☐ Other situation

**5. Have you been previously admitted to our hospital?**           ☐ Yes  ☐ No

**6. Upon admission, did you receive an identification wristband?**  ☐ Yes  ☐ No

**7. Upon admission to the ward/room, you were accompanied by:**

 ☐ Healthcare personnel

 ☐ Family members, friends, or neighbours

 ☐ I went alone

**8. You changed into hospital attire:**   ☐ At the cloakroom/wardrobe  ☐ In the room

**9. Upon admission, were you informed about:**

	YES	NO
Your rights and obligations as a patient		
Rules of conduct in the hospital		
Personal hygiene rules in the hospital		
The procedure for submitting suggestions and complaints		

**10. Please rate the quality of the following services**

	Unsatisfactory	Satisfactory	Good	Very good
Accommodation (facilities, associated spaces, hospital environment)				
Bed and bed linen				
Hospital clothing (gown, robe, slippers)				
Cleanliness of the hospital				
Sanitary facilities (bathrooms/toilets)				
Food/meals				
Meal service (manner of serving food)				

**11. Room cleaning is performed:**   ☐ As often as necessary  ☐ At least twice a day

**12. Please rate the quality of care provided by:**

	Unsatisfactory	Satisfactory	Good	Very good
Your attending physician				
Nurses				
Nursing auxiliaries/orderlies				
The courtesy and availability of the healthcare personnel				

**13. How satisfied are you with the cleanliness of the hospital?**

 ☐ Very dissatisfied  ☐ Dissatisfied  ☐ Satisfied  ☐ Very satisfied

**14. How satisfied are you with the activities and involvement of your physician?**

 ☐ Very dissatisfied  ☐ Dissatisfied  ☐ Satisfied  ☐ Very satisfied

**15. How satisfied are you with the activities and involvement of your nurse?**

 ☐ Very dissatisfied  ☐ Dissatisfied  ☐ Satisfied  ☐ Very satisfied

**16. How do you assess your communication with**

	Unsatisfactory	Satisfactory	Good	Very good
Your attending physician				
Nurses				
Nursing auxiliaries/orderlies				

**17. Did you receive clear information and understand the explanations regarding:**

	YES	NO
The treatment and investigations recommended by your attending physician		
The treatment plan		
The surgical/procedural/treatment risks		
The risks and possible errors related to the administration of medications and treatments you are receiving		
The diagnosis and the treatment to be administered		

**18. Do you consider that your rights as a patient have been respected, as specified in the regulations displayed in your ward/department?**  ☐ Yes  ☐ No

**19. Were you provided with spiritual assistance according to your religious faith?**

 ☐ Yes  ☐ No  ☐ Not applicable

**20. When attending medical investigations, consultations, or moving around the hospital, were you accompanied by designated personnel?**  ☐ Yes  ☐ No

**21. Upon admission, were you informed about the risk of falls/slipping within hospital premises?**

 ☐ Yes  ☐ No

**22. How do you rate the attitude of the UPU-SMURD / Emergency Reception staff during emergency registration (if you were admitted through this unit)?**   ☐ Unsatisfactory  ☐ Satisfactory  ☐ Good  ☐ Very good

**23. How do you rate the care received in the Intensive Care Unit (if applicable)?**

 ☐ Unsatisfactory  ☐ Satisfactory  ☐ Good  ☐ Very good

**24. Medication Safety and Clinical Care Standards**

	Yes, always	Yes, sometimes	No, never
Was the prescribed treatment regimen explained to you at discharge?			
Were you able to easily identify the medical personnel involved in your treatment?			
Was medication administration (oral, rectal, vaginal, or cutaneous route) carried out under the supervision of a nurse?			
Does the medical staff use disposable gloves at every patient contact?			

**25. You received your daily medications:**  ☐ All at once  ☐ Divided into separate doses

**26. Outcome Quality and Ethical Conduct**

	YES	NO
During your hospital stay, did you need to purchase medications or other medical supplies yourself?		
Is your health condition better after discharge?		
Were you informed about the estimated date of discharge?		
Would you recommend this hospital to a person close to you?		
Were you asked for money or gifts by physicians or nurses? If yes, do you wish to report to the Anti-Corruption Officer of the Ministry of Health that you were solicited for money or gifts?		

**27. If re-admission is necessary, would you choose this hospital?**  ☐ Definitely yes  ☐ Probably yes  ☐ Definitely no

**28. Your overall impression at discharge:**  ☐ Very dissatisfied  ☐ Dissatisfied  ☐ Satisfied  ☐ Very satisfied
